# Supplementary figures and images for: Trends in Incidence and Mortality of Kidney Cancer in a Northern Italian Province: An Update to 2020
Source: Biology (Basel). 2022 Jul 13;11(7):1048. doi: 10.3390/biology11071048 (PMC9311977; doi:10.3390/biology11071048)

Figure S1. Reggio Emilia Province: incidence and mortality trend for lung cancer by sex, years 2019-2020

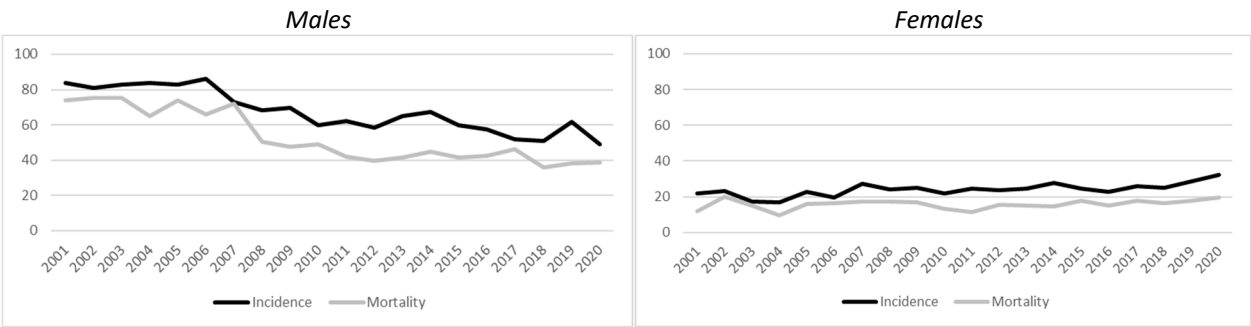

Supplement: Supplementary file 1 [file biology-11-01048-s001.zip › biology-1725536-supplementary.pdf]
